# An Observational Study of Paediatric Preoperative Transfusion Practice in a Resource-Limited Setting

**DOI:** 10.1007/s00268-021-06402-y

**Published:** 2022-01-10

**Authors:** Somy Charuvila, Tasmiah Tahera Aziz, Sarah E. Davidson, Ummay Naznin, Shiuly Sinha, Sabbir Ahmed, Kokila Lakhoo, Tahmina Banu

**Affiliations:** 1 Oxford University Global Surgery Group, Nuffield Department of Surgical Sciences, University of Oxford, Oxford, UK; 2 Chittagong Research Institute for Children Surgery (CRICS), Chittagong, Bangladesh

## Abstract

**Background:**

Paediatric anaemia is highly prevalent in low–middle-income countries and can negatively impact postoperative outcomes. Currently, there are no guidelines for the management of paediatric preoperative anaemia. To ensure optimal care in resource-limited settings: balancing the risks of anaemia and using resources such as blood transfusion, we first need to understand current practices. To address this, a joint UK–Bangladesh team conducted an observational study at a paediatric surgical centre in Bangladesh.

**Methods:**

A total of 464 patients ≤16 years who underwent elective and emergency surgery were categorised into major (351/464), moderate (92/464) and minor (21/464) surgery groups according to anticipated blood loss. Preoperative anaemia testing and transfusion was assessed retrospectively through patient notes.

**Results:**

Median age was 4 years and 73% were male. 32.5% (151/464) patients had preoperative blood testing for anaemia. 17.5% (81/464) children were transfused preoperatively. Of those children transfused, 40.7% (33/81) underwent transfusion solely based on visible signs of anaemia on clinical examination. Seventy-five percentage (36/48) of children who underwent transfusion after blood testing had haemoglobin ≥80 g/L. Major surgery category had the highest proportion of children who were transfused and tested for anaemia.

**Conclusion:**

A liberal transfusion approach is evident here. Discussion with local clinicians revealed that this was due to limitations in obtaining timely blood results and reduction in laboratory costs incurred by families when clinical suspicion of anaemia was high. Further research is needed to analyse the potential of using bedside haemoglobin testers in conjunction with patient blood management strategies to limit blood transfusions and its associated risks.

## Introduction

Paediatric anaemia is a major public health issue in developing countries owing to factors such as nutrition, infections and socioeconomic status. Anaemia is associated with higher mortality and organ dysfunction post-surgery [1]. It can increase length of hospital stay, blood transfusions and intensive care requirements [2].

Preoperative anaemia testing and treatment through patient blood management strategies, including restrictive transfusion, was discussed in our recent literature review [3]. To our knowledge, there is no guideline for paediatric preoperative anaemia testing and treatment in low-resource settings.

To address this, a global partnership between multidisciplinary professionals in the UK and Bangladesh was formed. To begin this process, an investigation of current practices was carried out at a major paediatric surgical centre in Bangladesh. This was done retrospectively from patient records and is described in this paper.

Prevalence of paediatric anaemia in Bangladesh has been quoted to be as high as 52% [4] and up to 24% [5] of children under 5 years in one study were identified to have severe anaemia [6]. Bangladesh’s economic status and high anaemia prevalence make it a suitable healthcare system for this study.

Health care in Bangladesh can be obtained from government and private sectors. At government institutions, patients do not incur consultation or operation fees. Their expenditure mainly involves investigations, medical supplies, food, accommodation and transport. Worldwide, 81 million people are estimated to experience financial catastrophe as a result of a surgical condition [7]. Health expenditure is significant, considering that 31.5% of the population in Bangladesh live below the poverty line and 70% live in areas with limited health care [8].

Chittagong Medical College Hospital (CMCH) in Bangladesh was chosen for this study as it is a government hospital with a paediatric surgery department serving a population of 30 million. It has a caseload of 2100 operations per year ranging from day cases to major surgeries. The centre has a long-standing partnership with a centre in the UK. It also has an outreach service facilitating surgical care for children in rural areas [9].

In the Chittagong region, anaemia prevalence in the 6- to 59-month age category has shown to be greater than 50% [10]. Despite the high prevalence, preoperative anaemia testing is not routine due to financial and infrastructural barriers. Table 1 indicates costs associated with blood tests and transfusion. Transfusion services such as screening, cross-matching and blood grouping are available all day. However, standard blood tests such as full blood count are done only from 8 am to 4 pm. Out-of-hours investigations can be obtained at higher costs from private centres. A recent study on COVID-19’s impact on economic loss in Bangladesh estimates that nearly 35% of workforce are daily wage earners receiving 272.2 Bangladeshi Taka (BDT) per day in the farm sector and 361.5 BDT per day in other sectors [11]. This estimation uses data from the most recent Household Income Expenditure Survey in Bangladesh (HIES) 2016–2017 conducted by the Bangladesh Bureau of Statistics. These figures may be subject to inflation and changes in living costs, and Table 1 nevertheless reflects the high cost of basic diagnostic services when compared to average daily wages.

**Table 1:** Cost comparison of blood diagnostic services at CMCH (government establishment) and at private centres in Bangladesh. Rates are Bangladeshi Taka (BDT). The average daily wage worker earns 272.2. BDT in the farm sector and 361.5 BDT in the non-farm sector

Investigation	CMCH (government hospital) costs in Bangladeshi Taka (BDT)	Cost range for private diagnostic centres in Bangladeshi Taka (BDT)
Full blood count (haemoglobin, total count, differential count and erythrocyte sedimentation rate)	150	(300–400)
Haematocrit alone	30	(150–200)
Routine preoperative screening blood test set (admitted patient)	250	(900–1200)
Routine preoperative screening blood test set (non-admitted patient)	500	(900–1200)
Blood grouping and rhesus typing	100	(300–400)
Cross-matching	100	(400–500)
Single transfusion set	250	250

Currently, there are no established criteria at CMCH to guide preoperative transfusion. When financial or logistical challenges limit preoperative anaemia testing, clinicians at CMCH use general medical knowledge of signs and symptoms of anaemia to clinically diagnose anaemia. Due to the high prevalence of untreated anaemia, clinicians often see signs of anaemia on examination. Conjunctival pallor, koilonychia, brittle nails, angular stomatitis, tachycardia in elective consultation and malnourished appearance are used to clinically diagnose anaemia. Symptoms such as fatigue, history of chronic illness or previous deworming treatment are also taken into consideration.

Most government diagnostic facilities are open only from 8 am to 4 pm. The high caseload and limited staff undertaking phlebotomy, transporting samples, preparation of laboratory equipment and the wait for an available automated analyser impact the productivity output from the laboratory between the constricted hours of 8 am to 4 pm. Moreover, results are not relayed after 4 pm due to lack of trained staff. Hence, it takes up to 24 h to obtain blood results in government hospitals like CMCH.

In cases where financial and logistical implications pose a challenge to anaemia testing, the anaesthetist and in some cases the senior surgical consultant initiate transfusion based on their clinical judgment. For those patients who undergo preoperative anaemia testing, clinicians use the maximum allowable blood loss calculation to guide transfusion [12].

$$ {\text{Maximum allowable blood loss}} = {\text{Estimated blood volume}}\;\frac{{\left( {{\text{Child's haematocrit}} - {\text{Minimum accepted haematocrit: i.e.}}\; 20\% \;{\text{in children}}} \right)}}{{{\text{Child's Haematocrit}}}} $$

If there is significant risk of blood loss (>7 mls/kg) as per the WHO Safe Surgery checklist, transfusion is requested even when there is low clinical suspicion or laboratory confirmation of anaemia. Other factors such as active bleeding, prolonged surgery, emergency surgery and coexisting malnourishment or haemoglobinopathy are also used to guide transfusion. Generally, a transfusion dose of 20 mL/kg of body weight is used.

Transfusion is also offered in non-urgent cases at CMCH. This is due to a backlog of elective surgeries and family resistance in postponing surgery for treatment of anaemia as they travel from rural areas and cannot afford further days off from work. Children are frequently brought to hospital when their chronic condition deteriorates and delaying their surgery can worsen outcomes.

To assess the current preoperative transfusion practice at CMCH, we conducted a retrospective study. Our aim was to investigate the proportion of children undergoing preoperative testing and transfusion when categorised according to surgical complexity. We focus on transfusion as the modality of anaemia treatment as it poses a significant challenge in terms of safety, logistics and cost.

## Methods

### Data collection

A total of 464 children aged 16 years or under (excluding neonates) who underwent elective and emergency surgery between 2014 and 2016 were included in this study. Data were retrospectively collected using a standardised proforma which included patient demographics, investigations, operative procedure, type of anaesthetic and details on preoperative anaemia testing and transfusion. This study did not impact clinical care of any patients and was strictly a retrospective observational study.

Data were collected by two research assistants at CMCH from October 2019 to December 2019. Video conferences were held between clinicians from the Bangladesh and UK teams throughout the study. Anonymised patient data from proformas were transferred to an excel spreadsheet which was analysed by researchers in the UK team.

### Data analysis

All patients were analysed according to major, moderate or minor surgery: this is an established criteria at CMCH and is used to clinically triage resources such as pre-op assessment, hospital beds and blood transfusion to patients. This categorisation is defined according to likelihood of performing the case under local or general anaesthesia and whether blood transfusion is likely based on the risk of blood loss.

For example, major surgery includes splenectomy, bowel surgery and solid organ tumour removal. These procedures are done under general anaesthetic and more likely to necessitate transfusion. Moderate surgery includes hernia repair and cystoscopic procedures. Examples of minor surgery include circumcision and keloid excision which are generally low risk, performed under local anaesthetic and are very unlikely to require transfusion.

Statistical significance between the number of patients tested for anaemia in each group was assessed using the Chi-squared (*χ*^2^) test. Proportion of patients who underwent preoperative blood transfusion was also assessed in these groups.

For those patients who underwent preoperative anaemia testing, we categorised them into two groups:

*Transfused versus non-transfused* For those patients who underwent preoperative anaemia testing, we assessed the mean difference in preoperative haemoglobin values between those transfused and not. Statistical significance between the mean preoperative haemoglobin of these two groups of patients was analysed using the t-test.

## Results

### Patient demographics

Table 2 shows patient demographics.

**Table 2:** Patient demographics

	*n*	%
Total	464	
*Age*		
Median	4.25 years	
Range	2 days to 16 years	
Children <1 year	90	19
Children <1 month	13	2.8
*Sex*		
Female	121	26
Male	341	73
Disorder of sex development	2	0.4
*Type of surgery*		
Major	351	76
Moderate	92	20
Minor	21	4
*Type of anaesthetic*		
General	447	96.3
Spinal	8	1.7
Sedation	6	1.3
Local	3	0.6
*Transfusion status*		
Transfused	81	17.5
Non-transfused	383	82.5
*Preoperative anaemia testing*		
Tested	151	32.5
Non-tested	313	67.5

Table 3 shows the range of surgical procedures included in this study.

**Table 3:** Surgical procedures underwent by patients in this study

Surgical procedure	*N*
Laparotomy (trauma, obstruction, tumour, splenectomy)	99
Urethroplasty/hypospadias/chordee release	67
Cystoscopy—diagnostic and procedural	47
Cleft lip/cleft palate repair	44
Anoplasty/genitoplasty	42
Burn contracture release/graft	24
Teratoma/parotid/cystic hygroma/goitre removal	24
Haemangioma excision/sclerotherapy	20
Pull through procedure for Hirschsprung’s	20
Hernia repair	18
Minor wound debridement/abscess/keloid or cyst removal	13
Nephrostomy	6
Anal fistula procedure	5
Pyloromyotomy	5
Circumcision	4
Cystostomy formation	4
Diagnostic laparoscopy	4
Urethral fistula repair	4
Endoscopy	3
EUA	3
Ureteric implantation	3
Foreign body chest wall	2
Syndactyly repair	2
Orchidopexy	1

### Preoperative transfusion practice

**Fig. 1:**
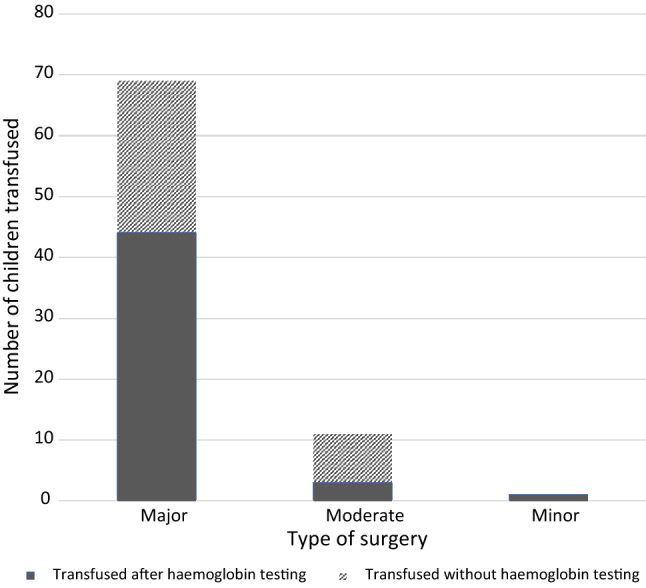
Comparison of the transfusion practice according to the type of surgery

Table 4 and Fig. 1 outline the transfusion practice by surgical category.

**Table 4:** Data on the transfusion practice according to the type of surgery

	Major	Moderate	Minor
Total patients (*n* = 464)	351	92	21
Total transfused (*n* = 81)	69	11	1
Transfused after haemoglobin testing (*n* = 48)	44	3	1
Transfused without haemoglobin testing (*n* = 33)	25	8	0

Overall, 81 of the total 464 children were transfused of whom 85% (69/81) were in the major surgery category. One patient in the minor surgery category with a recorded Hb of 120 g/L underwent transfusion for faecaloma extraction. This outlier was due to anticipated blood loss from the inflamed colonic wall. 40.7% (33/81) of patients underwent transfusion without prior blood test confirming anaemia.

**Fig. 2:**
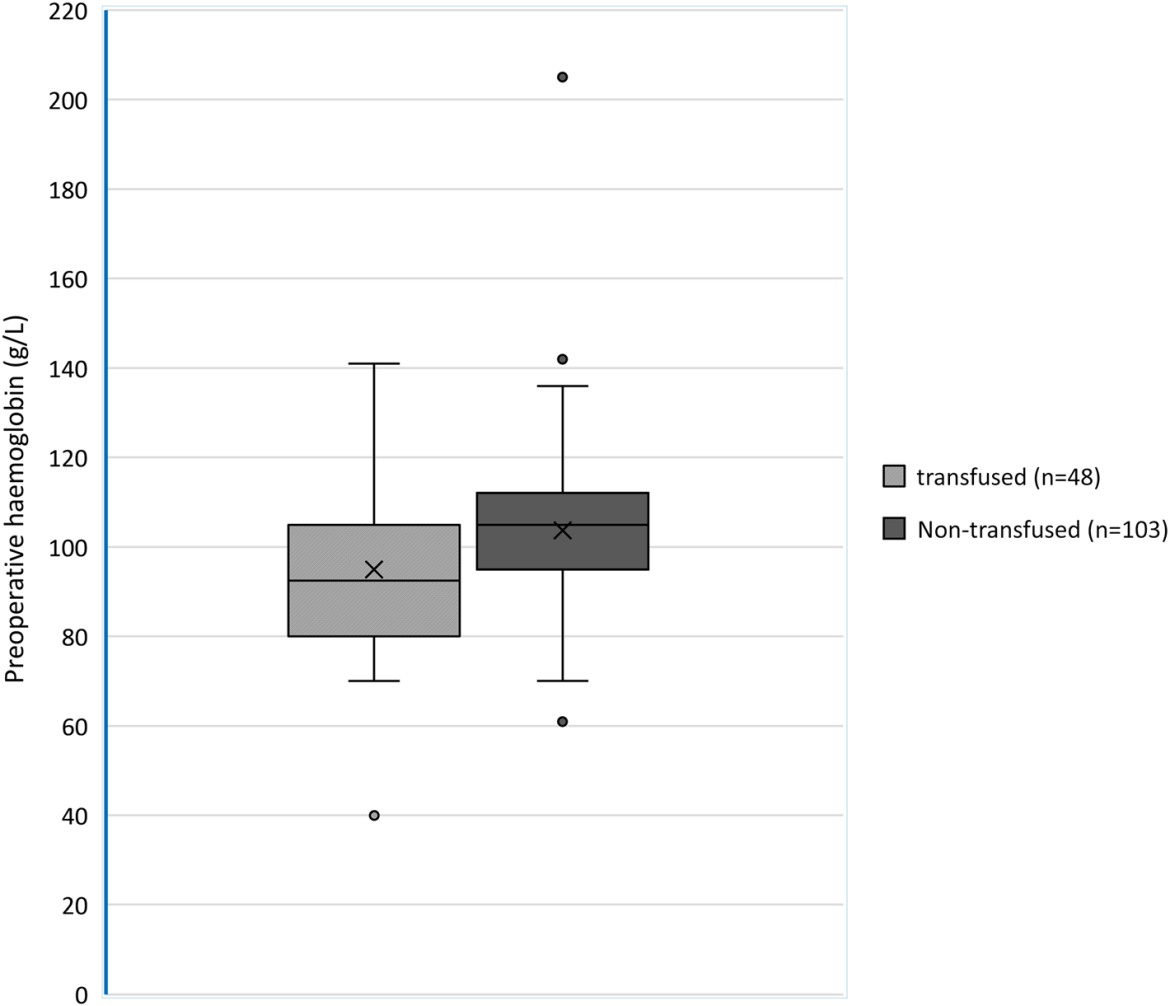
Preoperative haemoglobin values in transfused versus non-transfused groups where *n* indicates the number of patients in each group who have recorded haemoglobin values

Only 151 out of the total 464 patients had preoperative anaemia testing. Among these 151 children, we compared the haemoglobin values of patients who were transfused vs non-transfused. This is displayed in the box and whisker plot chart in Fig. 2.

The mean preoperative haemoglobin of 94.9 g/L in the transfused group is lower than that of the non-transfused group 103.7 g/L. This difference is statistically significant (*p* = 0.003) as per the t-test.

Patients who were transfused had lower haemoglobin values. However, the haemoglobin threshold which guided the decision to transfuse is not evident. Clinical context was used to determine the need for transfusion. Indeed, patients above the World Health Organization anaemia threshold ranges were also transfused [13] (Fig. 2). Among the 48 patients transfused after a blood test, 75% (36/48) had an Hb ≥ 80 g/L as indicated by their lower quartile mark in Fig. 2.

### Preoperative anaemia testing practice

Majority of patients tested for anaemia fell into the major category (Table 5). *Χ*^2^ test indicates the testing rates among the different groups are statistically significant (*p* = 3.2637 × 10^−5^).

**Table 5:** Testing rates according to category of surgery

	Haemoglobin tested (*n* = 151)	Haemoglobin non-tested (*n* = 313)
Major (*n* = 351)	133	218
Moderate (*n* = 92)	12	80

Of the 464 patients in this retrospective analysis, there was 0% mortality at 6 months postoperatively.

## Discussion

This study has highlighted disparities in care owing to financial, infrastructural and cultural reasons. This study had a significantly high percentage of males (73%). Studies in Bangladesh and south Asia have shown that the sex of a child can influence parental health seeking behaviour and adherence to treatment [14–16]. However, these factors were not investigated in this study and hence we cannot determine the reasons for this difference.

Majority of children (76%) underwent major surgery. CMCH being a large tertiary centre receives many complex referrals from less equipped hospitals. The high percentage of major surgery also potentially reflects the culture in rural communities where parents do not seek surgical intervention until significant deterioration of their child’s condition.

Here, 85% of those transfused underwent major surgery which is usually associated with higher risk of blood loss and coexisting medical comorbidities. Forty percentage of patients transfused had not undergone prior blood testing for anaemia, again with a predominance in the major surgery group. In patients with visible features of anaemia undergoing emergency surgery with high risk of blood loss, clinicians did not request preoperative anaemia testing due to the long waiting time for blood results (up to 24 h). Clinicians were also significantly influenced by families’ ability to cover testing costs.

Seventy-five percentage of the patients who underwent haemoglobin testing prior to transfusion had a haemoglobin >80 g/L. Discussion with clinicians outlines reasons such as anticipated volume of blood loss and comorbidities of the patient. Similarly, a Chinese study of 1506 children undergoing intraoperative transfusion found that 45.3% of those transfused had an Hb >80 g/L and 28.8% had Hb >100 g/L. This was attributed to haemodynamic parameters, body size, clinician preference and lack of paediatric perioperative guidelines [17].

Although preoperative anaemia carries negative outcomes, the haemoglobin threshold to warrant postponement of surgery is unknown. Evidence from the landmark Canadian paediatric ICU trial involving 637 children showed that in stable critically ill children, a threshold of 70 g/L can reduce the transfusion requirements without being associated with adverse outcomes when compared to a liberal threshold of 95 g/L [18]. A Danish study of 210 adolescents undergoing scoliosis surgery also advocated a threshold of 70 g/L [19].

A review of six studies also concluded that a threshold of 69.3 g/L for children admitted to ICU with burns, sepsis or after general and cardiac surgery was safe (excluding cyanotic heart disease) [20]. Importantly, these studies on restrictive transfusion were conducted in settings well resourced for monitoring and testing. Additionally, they have been done in the context of the critical care and cannot be translated directly to the preoperative setting.

The likelihood of over-transfusion in this study is high because clinical assessment of anaemia can be subjective. Blood transfusion carries several risks including infections, febrile reactions, donor mismatch, lung injury and anaphylaxis. Clinicians at CMCH reported febrile reactions, urticarial, allergic reactions and fluid overload in the last 5 years.

Screening donated blood for HIV, HBV, HCV, syphilis and malaria is mandatory in Bangladesh. However, studies in Bangladesh and India have shown the presence of hepatitis B in donated blood that underwent pre-transfusion screening [21–23]. This is potentially due to lack of trained staff and highly sensitive modalities of screening.

Transfusion also poses logistical challenges. Due to the high prevalence of blood borne infections among paid donors, the *Safe Blood Transfusion* initiative was implemented in Bangladesh, which led to a sharp decrease in the number of paid donors from 70 to 10% in the early 2000s [24]. Hence, blood banks depend on blood donation from relatives, voluntary organisations and hospital staff (when there is a lack of donors and families are unfit to donate).

Blood for donation is usually arranged on demand. For non-urgent cases at CMCH, it can take up to 18 h to carry out donor selection, screening, cross-matching and organising equipment. Written informed consent is taken prior to transfusion.

The health risks and costs highlight the need for titration of transfusion to haemoglobin levels. Bedside point-of-care (POC) haemoglobin testing can increase preoperative anaemia testing. POC testing for perinatal HIV, syphilis and malaria infections have been shown to be very useful in resource-limited settings [25]. POC anaemia testing has been demonstrated to be of comparable accuracy to standard methods [26]. A meta-analysis showed that diagnosing anaemia via POC testing was more accurate than clinical assessment alone [27].

**Fig. 3:**
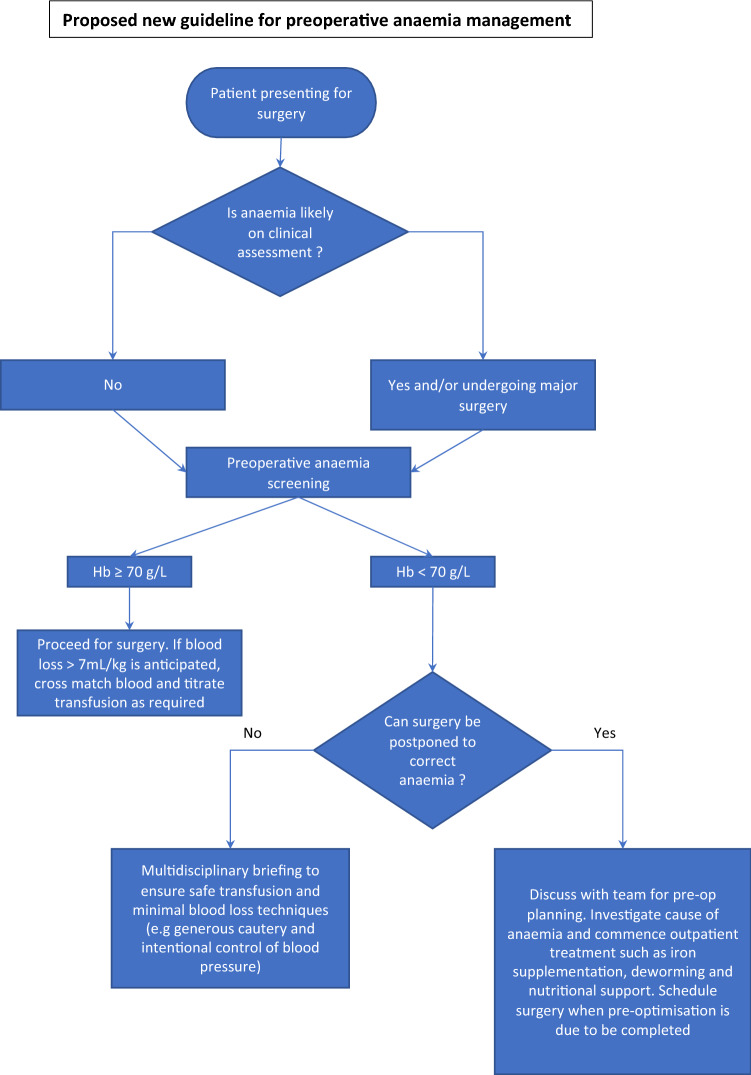
Proposed new guideline for future prospective study. This guideline was created in response to the findings of this retrospective study

We propose a prospective study on the clinical impact of POC anaemia testing alongside a restrictive transfusion approach. To conduct the prospective study, we have created a new decision-making guide (Fig. 3). This guide involves a multidisciplinary decision-making approach in aspects such as restrictive blood transfusion, intentional blood pressure control, generous cautery, judicious use of tourniquets and tranexamic acid to reduce bleeding on a case-by-case basis.

Tranexamic acid is a widely available drug at CMCH and is currently considered on an individual basis in the context of trauma and surgery involving significant risk of blood loss (>7 mls/kg). Tranexamic acid has been recommended in paediatric trauma and major surgery as there is evidence that it reduces perioperative blood loss and need for transfusions [28].

This new proposed guide suggests postponing elective surgery in certain cases, after careful consideration of the patient’s clinical picture and financial circumstances (such as travel and accommodation costs for patients from rural regions). Where postponing surgery will not impact long term health, expedited surgery can be arranged after optimisation of haemoglobin level.

As part of this prospective study, educational awareness regarding transfusion risks and patient blood management strategies is being given to clinicians at CMCH and rural referring hospitals through existing outreach programs.

## Conclusion

This study has highlighted the complexity of the decision-making process in preoperative anaemia assessment and management in a resource-limited setting. Discussions with local clinicians have given insight into the challenges presented by lack of affordable rapid anaemia testing.

Restrictive transfusion in conjunction with increased preoperative anaemia testing may be beneficial in resource-limited settings. We aim to analyse this in a prospective study.
